# Clinical Utility of a Digital Dermoscopy Image-Based Artificial Intelligence Device in the Diagnosis and Management of Skin Cancer by Dermatologists

**DOI:** 10.3390/cancers16213592

**Published:** 2024-10-24

**Authors:** Alexander M. Witkowski, Joshua Burshtein, Michael Christopher, Clay Cockerell, Lilia Correa, David Cotter, Darrell L. Ellis, Aaron S. Farberg, Jane M. Grant-Kels, Teri M. Greiling, James M. Grichnik, Sancy A. Leachman, Anthony Linfante, Ashfaq Marghoob, Etan Marks, Khoa Nguyen, Alex G. Ortega-Loayza, Gyorgy Paragh, Giovanni Pellacani, Harold Rabinovitz, Darrell Rigel, Daniel M. Siegel, Eingun James Song, David Swanson, David Trask, Joanna Ludzik

**Affiliations:** 1 Department of Dermatology, Oregon Health and Science University, Portland, OR 97239, USA; witkowsa@ohsu.edu (A.M.W.); greiling@ohsu.edu (T.M.G.); leachmas@ohsu.edu (S.A.L.); nguyekho@ohsu.edu (K.N.); ortegalo@ohsu.edu (A.G.O.-L.); 2 Department of Dermatology, University of Illinois-Chicago, Chicago, IL 60637, USA; jbursh2@uic.edu; 3 Ironwood Dermatology, Tucson, AZ 85737, USA; mchristophermd@gmail.com; 4 Cockerell Dermatopathology, Dallas, TX 75230, USA; ccockerell@dermpath.com; 5 Department of Dermatology & Cutaneous Oncology, University of South Florida, Tampa, FL 33612, USA; lcorrea1@usf.edu (L.C.); grichnik@usf.edu (J.M.G.); 6 Las Vegas Dermatology, Las Vegas, NV 89144, USA; dcotter@lvderm.com; 7 Department of Dermatology, Nashville VA Medical Centers and Vanderbilt University, Nashville, TN 37232, USA; darrel.ellis@vumc.org; 8 Bare Dermatology, Dallas, TX 75235, USA; afarberg@barederm.com; 9 Department of Dermatology, University of Connecticut School of Medicine, Farmington, CT 06030, USA; grant@uchc.edu; 10 Department of Dermatology, University of Florida College of Medicine, Gainesville, FL 32606, USA; 11 Department of Dermatology and Dermatopathology, University of Texas Medical Branch, Galveston, TX 77555, USA; aqlinfan@utmb.edu; 12 Dermatology Service, Memorial Sloan Kettering Skin Cancer Center, Hauppauge, NY 11788, USA; marghooa@mskcc.org; 13 Skin Pathology Associates, Delray Beach, FL 33446, USA; etan.marks@adcsclinics.com; 14 Department of Dermatology, Roswell Park Comprehensive Cancer Center, Buffalo, NY 14263, USA; gyorgy.paragh@roswellpark.org; 15 Department of Dermatology, La Sapienza University, Rome 00161, Italy; giovanni.pellacani@uniroma1.it; 16 Skin and Cancer Associates, Plantation, FL 33324, USA; hrabinovitz@augusta.edu; 17 Department of Dermatology, NYU Grossman School of Medicine, New York, NY 10016, USA; darrell.rigel@nyulangone.org; 18 Department of Dermatology, SUNY Downstate Health Sciences University, Brooklyn, NY 11203, USA; daniel.siegel@downstate.edu; 19 Frontier Dermatology, Salem, OR 97302, USA; esong812@gmail.com; 20 Department of Dermatology, Mayo Clinic Arizona, Scottsdale, AZ 85259, USA; swanson.david@mayo.edu; 21 Skin Cancer & Dermatology Center of Southern Oregon, Medford, OR 97504, USA; teresa@traskskindr.com

**Keywords:** melanoma, basal cell carcinoma, squamous cell carcinoma, skin cancer, atypical nevi, dermoscopy, dermatoscopy, artificial intelligence, machine learning, convolutional neural network

## Abstract

Background: Patients with skin lesions suspicious for skin cancer or atypical melanocytic nevi of uncertain malignant potential often present to dermatologists, who may have variable dermoscopy triage clinical experience. Objective: To evaluate the clinical utility of a digital dermoscopy image-based artificial intelligence algorithm (DDI-AI device) on the diagnosis and management of skin cancers by dermatologists. Methods: Thirty-six United States board-certified dermatologists evaluated 50 clinical images and 50 digital dermoscopy images of the same skin lesions (25 malignant and 25 benign), first without and then with knowledge of the DDI-AI device output. Participants indicated whether they thought the lesion was likely benign (unremarkable) or malignant (suspicious). Results: The management sensitivity of dermatologists using the DDI-AI device was 91.1%, compared to 84.3% with DDI, and 70.0% with clinical images. The management specificity was 71.0%, compared to 68.4% and 64.9%, respectively. The diagnostic sensitivity of dermatologists using the DDI-AI device was 86.1%, compared to 78.8% with DDI, and 63.4% with clinical images. Diagnostic specificity using the DDI-AI device increased to 80.7%, compared to 75.9% and 73.6%, respectively. Conclusion: The use of the DDI-AI device may quickly, safely, and effectively improve dermoscopy performance, skin cancer diagnosis, and management when used by dermatologists, independent of training and experience.

## Simple Summary

Patients with skin lesions suspicious for skin cancer or atypical melanocytic nevi of uncertain malignant potential often present to dermatologists, who may have variable dermoscopy triage clinical experience. We evaluated the clinical utility of a digital dermoscopy image-based artificial intelligence algorithm (DDI-AI device) on the diagnosis and management of skin cancers. Thirty-six United States board-certified dermatologists evaluated 50 cases with clinical images and DDI of the same skin lesions (25 malignant and 25 benign), first without and then with knowledge of the DDI-AI device output. We identified that when using the DDI-AI device, management sensitivity, management specificity, diagnostic sensitivity, and diagnostic specificity all increased over baseline use of clinical images and dermoscopy images. Hence, the use of the DDI-AI device may quickly, safely, and effectively improve dermoscopy performance, skin cancer diagnosis, and management when used by dermatologists, independent of training and experience.

## 1. Introduction

Skin cancer is the most prevalent form of malignancy in the United States (U.S.), and its incidence is increasing more than any other cancer [1,2]. Early identification and diagnosis are critical to reduce morbidity and improve prognosis, especially for malignant melanoma (MM) [3,4]. Dermatologists evaluate, and if necessary, biopsy, patients with skin lesions that are suspicious for skin cancer, or atypical melanocytic nevi of uncertain malignant potential (AMNUMP), to determine diagnosis. Traditionally, the ABCDE criteria (asymmetry, irregular borders, multiple colors, diameter > 6 mm) have been the standard for the visual evaluation of target skin lesions of concern (TSLCs) and diagnosis of skin cancers and AMNUMPs [5,6]. In recent years, several advanced in vivo technological innovations have become available to aid clinician biopsy selection accuracy. These technologies include dermoscopy, laser-based tools (reflectance confocal microscopy (RCM), optical coherence tomography (OCT), multispectroscopy), electrical impedance spectroscopy (EIS), elastic scattering spectroscopy (ESS), and 2-gene expression profiling (2-GEP). When used appropriately, they have been shown to improve the accuracy of skin cancer diagnosis and may improve health outcomes [7,8,9,10,11,12,13,14,15,16,17,18,19,20,21]. 

After naked-eye visual inspection, dermoscopy has become the standard of care for first-level triage and has proven to augment triage of TSLCs when applied by a dermatologist experienced in dermoscopy [22,23,24]. Dermoscopy is also used to direct selection of secondary triage tools like RCM, OCT, and spectroscopy due to its ability to provide context to a TSLC’s surface and subsurface structures, including colors associated with potential malignancy that are not visible with naked eye evaluation. Usage of dermoscopy has become an integral part of dermatology residency training worldwide, and diagnostic accuracy is significantly dependent upon the dermatologist’s education and experience, which can lead to variations in triage performance. Therefore, an innovative dermoscopy expert trained Software as a Medical Device (SaMD) dermoscopy image-based digital artificial intelligence algorithm (DDI-AI device) has the potential to improve traditional handheld dermoscopy triage, may reduce subjective dermoscopic evaluation in beginner to intermediate dermoscopists, and may augment objective decision-making related to TSLCs. Enhancing dermoscopy performance presents an opportunity to identify more skin cancers, improve earlier detection, and increase triage specificity.

In this clinical utility study, we investigated the use of a DDI-AI device that helps to detect skin cancers (MM, basal cell carcinoma (BCC), squamous cell carcinoma (SCC)) and AMNUMPs. The DDI-AI device was developed to be used as a stand-alone tool for rapid evaluation and triage of TSLCs presenting to a dermatology office that have either been previously identified by the patient, dermatology resident, or dermatology specialist during a spot check or total body skin exam. The intended use of the DDI-AI device is to improve bedside skin cancer detection performance. The DDI-AI device is not a diagnostic tool and is not intended to be used as a full body mole scanner or replace current methods of initial TSLC identification (ABCDE criteria, “ugly duck sign”, handheld dermoscopy [25].

We compared the diagnostic and management performance (sensitivity, specificity, and ROC) of board-certified dermatologists with and without the use of the DDI-AI device for detecting skin cancers and AMNUMPs. This study hypothesizes that the use of a stand-alone DDI-AI device can help dermatologists improve their bedside dermoscopy performance.

## 2. Materials and Methods

### 2.1. Digital Dermoscopy Image-Based Artificial Intelligence System (DDI-AI Device)

The investigative device received FDA Breakthrough Status Designation (FDA #Q211049; Sklip System; 22 June 2021, Sklip Inc., Lake Oswego, OR, USA) and is a stand-alone SaMD DDI-AI device that employs evaluation of the modified dermoscopy three-point checklist (MD3PC) criteria in conjunction with an internationally recognized dermoscopy expert image dataset annotation and supervised machine learning approach of convolutional neural networks (CNNs) that helps to detect skin cancers (MM, BCC, SCC) and AMNUMPs [11,21]. The DDI-AI device accepts DDI input generated from the following FDA class 1 registered medical devices: Sklip dermatoscopes (Original, PRO) (Sklip, Inc., Lake Oswego, OR, USA) and Heine dermatoscopes (DELTA 30 PRO, DELTA 30, DELTAone) (Heine USA Ltd., Dover, NH, USA). The DDI-AI device integrates into currently available smartphones and tablets with iPhone mobile operating system (iOS) and Android mobile operating system through an associated smartphone app shell (Sklip, app, Sklip, Inc., Lake Oswego, OR, USA).

For dermatologists, the DDI-AI device is intended to augment existing TSLC selection practices by providing the user with additional objective dermoscopy information that may be used in context for further patient workup and management decisions. The device classifies DDI input as either positive (“SUSPICIOUS”) or negative (“UNREMARKABLE”), where positive skin lesions warrant further workup (independent clinician choice to perform a biopsy, short-term monitor, or other diagnostic testing) and negative skin lesions warrant self-monitoring by the patient until the TSLC changes or evolves, where it would require a repeated examination or immediate second opinion by another dermatologist, where it may be biopsied. If an “ERROR” output is received on three consecutive attempts on the same TSLC (i.e., inadequate immersion liquid, artifacts are present, or incorrect hardware is used), information is provided to the user to not consider the DDI-AI device output in further workup of the TSLC in question and continue management independent of the DDI-AI device output. 

### 2.2. Study Test Lesions (Cases)

Fifty cases were selected and included 25 malignant lesions and 25 benign lesions. All malignant lesions and nine benign lesions were previously biopsied and diagnosed by a board-certified dermatopathologist to determine ground truth. Sixteen unbiopsied benign lesions were classified by dermatologists (readers: dermoscopy experts) using a CI and DDI, and ground truth was determined when at least two of three readers had concordant management and diagnosis. Characteristics of the test skin lesions are presented in Table 1. 

Study cases were randomly selected to match the prevalence of lesions in similar clinical utility device studies [18]. Notably, like other studies, actinic keratoses were not treated as malignant for the purposes of this classification, and AMNUMPS (severely atypical melanocytic nevi) were considered to need further evaluation by a dermatologist (including consideration for 0.5 mm margin excision). For each case, a digital clinical image, DDI, patients’ age, and anatomic location of the TSLCs were included. All patient clinical images and DDI used in this study were from retrospective chart reviews where all protected health information (PHI) was de-identified in accordance with the HIPAA Privacy Rule.

The 50 target skin lesions evaluated by dermatologists were dispersed across the body and had a mixture of pigmentation, color, surfaces, and textures; 26% (13/50) were melanocytic, 68% (34/50) were biopsied, and there was a range of final lesion etiologies. Previous clinical study performance of the DDI-AI device reported to the FDA was 94.5% sensitivity, 83.3% specificity, and 16.7% false-positive rate (FPR) as part of the device FDA Breakthrough Status Designation. The device performance for the lesions in this study was fixed at 100.0% sensitivity, 68.0% specificity, and 32.0% FPR. The malignant and benign subgroups were based on predefined proportions applicable to dermatology care, and enrolled lesions matching these criteria were selected from the clinical study dataset to match the relevant diagnoses [18].

### 2.3. Board-Certified Dermatologist Evaluations

**Figure 1:**
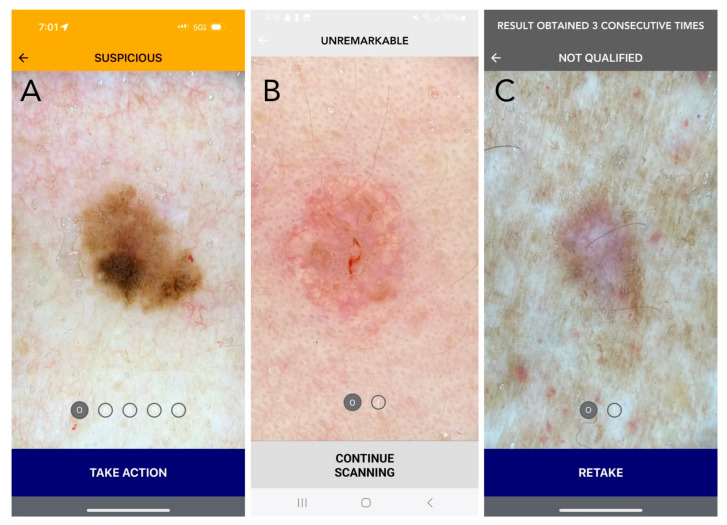
Current DDI-AI device outputs and labeling (Apple iPhone 15 PRO: (**A**,**C**); Samsung A14: (**B**)). (**A**) SUSPICIOUS (melanoma in situ): Positive (MD3PC) with moderate to high concern for malignancy. Recommended action: Additional evaluation is recommended, as determined appropriate by the medical professional. For a dermatologist, this may be a biopsy, or referral to another dermatologist for a second opinion. If the dermatologist chooses not to biopsy, it is strongly recommended that they document the DDI by uploading the DDI-AI output result to the patient electronic medical record (EMR). For patient safety, in the setting of biopsy avoidance, the DDI-AI recommends the user to complete a DDI spot check follow-up within a 3-month period. The dermatologist should also explain the patient to communicate earlier in the event a change is observed in mole size, shape, or color at any time during the 3-month period. (**B**) UNREMARKABLE (sebaceous hyperplasia): No immediate concern, limited or no positive MD3PC, with low concern for malignancy. Recommended action: The dermatologist should consider documenting the DDI by uploading the DDI-AI output result to the patient EMR. Recommend a DDI spot check follow-up within a 6-month period. The clinician should also explain the patient to communicate earlier in the event a change is observed in mole size, shape, or color at any time. (**C**) ERROR (dermatofibroma): The image does not meet DDI-AI technical inclusion criteria and an assessment of the uploaded DDI cannot be made. This result may be returned to the user when the image is not the right sort of item, inadequate immersion oil is used, or when the image quality is insufficient. Recommended action: If an “ERROR” result is obtained, the labeling information explains how to properly retake DDI with a maximum of three attempts per target lesion. If there are three failed consecutive attempts on the same TSLC, DDI-AI labeling instructs the user to not consider the DDI-AI output in their decision-making process and use their independent clinical judgement. Disclaimer: The use of DDI-AI is not intended to issue a final diagnosis and is not a total body mole scanner.

A total of 36 United States board-certified dermatologists, composed of 31 general dermatologists (29 MDs, 2 DOs) and six dermatopathologists (5 MDs, 1 DO), provided informed consent to participate in the study. All participants were provided instructions on how to interpret the DDI-AI device outputs before moving forward with their participation in the study (Figure 1) and were blinded to the study distribution of benign and malignant skin lesions. Participant experience and demographic information are presented in Table 2.

The study was conducted in four phases; in the first three phases, participants evaluated all 50 TSLCs (cases) independently and in random order. In the first phase, each participant evaluated the cases using clinical images only. In the second phase, the participants evaluated the same cases with the addition of a DDI image. Participants were blinded to the DDI-AI device outputs in phases one and two. For each case, participants completed a questionnaire using an IRB-approved secure cloud-based survey (Qualtrics, Provo, UT, USA) about their suggested management of the case (biopsy, monitor, or referral to a dermatology office), diagnosis (malignant or benign), and their level of confidence regarding their respective management and diagnostic decisions (scale 0–3, where 0 = no confidence, 1 = slight confidence, 2 = moderate confidence, and 3 = high confidence).

**Figure 2:**
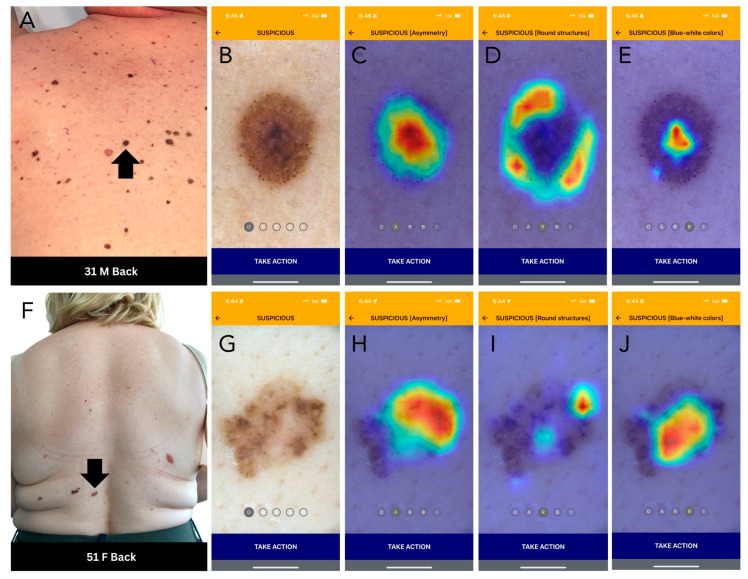
DDI-AI device outputs. (**A**) Clinical image of a melanoma (black arrow). (**B**) DDI-AI device suspicious output. (**C**–**E**) DDI-AI device transparent heatmap outputs based on the possible presence of modified dermoscopy three-point checklist criteria (MD3PC), presented individually to the user. (**F**) Clinical image of a basal cell carcinoma (black arrow). (**G**) DDI-AI device suspicious output. (**H**–**J**) DDI-AI device transparent heatmap outputs.

In the third phase, participants were asked to evaluate the same cases in a different randomized order; however, this time with the additional knowledge of the DDI-AI device output (suspicious, unremarkable, or error). In the fourth phase, participants were asked to evaluate only those cases that were classified by the DDI-AI in phase three as suspicious, including 16 malignant cases (omitting nine SCCs due to absence of intended DDI-AI device transparent heatmap use) and four false positive benign cases (two seborrheic keratosis, one blue nevus, and one atypical melanocytic nevus with moderate atypia) in a different randomized order, with the additional knowledge of the DDI-AI device transparent heatmap outputs highlighting where the DDI-AI device identified the presence of a possible MD3PC feature (red: high likelihood, orange/yellow: moderate likelihood, green/teal: low likelihood, purple: very little-to-no likelihood) (Figure 2). For phases two through four, participants were requested to answer an additional fifth question about which MD3PC feature (asymmetry and/or atypical pigment network, round structures, or blue–white–grey–violet color(s)) was most predominant (when applicable). Each participant answered a total of 798 questions to qualify as a completed study survey. All participant survey responses were exported securely and evaluated by a third-party statistician.

Each performance parameter, including sensitivity and specificity, was calculated using the total number of evaluations (n = 1800; 50 lesions × 36 dermatologists). A similar approach was followed to determine and compare across study phases the management decisions, where the sensitivity was defined as the probability of correctly deciding to further evaluate a lesion through a recommendation to obtain a potential biopsy or second opinion (referral) from another dermatologist, given the suspicion that the lesion was malignant, whereas specificity was defined as the probability of correctly deciding not to further evaluate the lesion (i.e., monitor), given its likely benign (unremarkable) nature. To accommodate near-extreme proportions, the corresponding 95% confidence intervals (CIs) for sensitivity and specificity were calculated using the Wilson method [26]. The effect of the level of confidence of the participants on their respective diagnostic and management sensitivity and specificity were assessed using binary multiple logistic regressions on the correct clinicopathologic diagnosis (malignant or benign). The effects of correct participant management, their respective confidence, and the predominant MD3PC features alluding to the diagnosis were calculated as adjusted odds ratios [27]. The logistic regression models were additionally used to produce the ROC analysis and to calculate AUC statistics for the diagnostic and management performance of participants during each study phase. The differences in AUC values were compared using the paired bootstrapping method (where applicable). Inter-dermatologist variability in the level of confidence regarding their management and diagnosis decision with and without the DDI-AI device was assessed using a Cohen’s unweighted kappa statistic [28]. A two-sided alpha level of less than 0.05 was considered the threshold for statistical significance for all comparisons. All statistical analyses were performed in R Studio (Posit Software, PBC, Version 2024.04.2+764, 2024), with the use of packages tidyverse, pROC, binom, irr, and lpSolve, where applicable.

## 3. Results

The 36 participants represented a diverse group of dermatologists in a broad range of dermatology practice types (Table 2). 

The unadjusted management sensitivity of the dermatologists using the DDI-AI device was 91.1% (95% CI [89.1–92.8%]), compared to 84.3% (95% CI [81.8–86.6%]) with DDI, and 70.0% (95% CI [66.9–72.9%]) with clinical images only. The management specificity using the DDI-AI device was 71.0% (95% CI [68.0–73.9%], compared to 68.4% (95% CI [65.3–71.4%]) with DDI, and 64.9% (95% CI [61.7–67.9%]) with clinical images. The unadjusted diagnostic sensitivity of the dermatologists using the DDI-AI device was 86.1% (95% CI [83.7–88.2%]), compared to 78.8% (95% CI [76.0–81.3%]) with DDI, and 63.4% (95% CI [60.2.–66.5%]) with clinical images only. The diagnostic specificity using the DDI-AI device was 80.7% (95% CI [78.0–83.1%], compared to 75.9% (95% CI [73.0–78.6%]) with DDI, and 73.6% (95% CI [70.6–76.3%]) with clinical images (Table 3). Balanced accuracy (TP + TN/all cases) of dermatologist management performance was 67.4% with clinical images, 76.4% with DDI, and 81.1% with the DDI-AI device.

**Figure 3:**
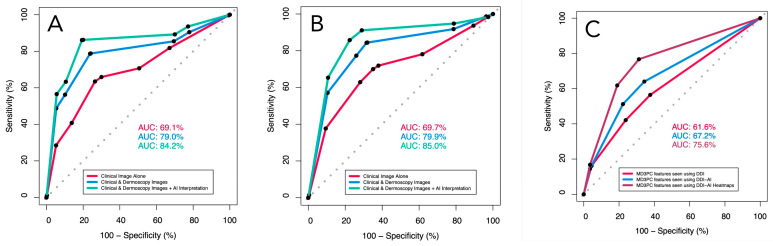
The receiver operator characteristics (ROC) for dermatologist (**A**) diagnostic sensitivity and specificity, (**B**) management sensitivity and specificity, both adjusted for participant confidence in their decision (none, slight, moderate, high), and (**C**) MD3PC-specific features for detecting malignancy. Red line (dermatologists using DDI evaluated with clinical experience), blue line (same dermatologists using the DDI-AI device output), and green line (dermatologists using the DDI-AI heatmaps).

In the ROC analysis, the management performance of the dermatologists adjusted for operator confidence improved significantly between viewing clinical images alone and using DDI (AUC = 69.7%, 95 CI [67.3–72.1] vs. AUC = 79.9% [77.8–82.0]; (*p* < 0.00001); their performance improved further (AUC = 85.0% (95% CI [83.2:86.8]) when switching from DDI to DDI-AI (*p* = 0.00033). Similarly, their diagnostic performance increased significantly from using clinical images alone (AUC = 69.1%, 95 CI [66.7–71.6]) to using DDI (AUC = 79.0, 95% CI [76.8–71.6]; *p* < 0.00001), and to using the DDI-AI device (AUC = 84.2%, 95% CI [82.3–86.1]; *p* < 0.00001 (Table 4) (Figure 3). 

Reflective of this relationship, the odds of choosing to proceed with biopsy (or refer to another dermatologist for second opinion) over monitoring when the blinded outcome was a malignant lesion improved from viewing clinical images alone (OR = 4.27; 95% CI [3.50:5.22]) to using DDI (OR = 12.9; 95% CI [10.2:16.4]), to using the DDI-AI device (28.8; 95% CI [21.8:38.4]), while adjusting for operator confidence. Similarly, the odds of correctly identifying a malignant lesion improved with the addition of the DDI-AI device interpretation. Importantly, the predominant MD3PC-specific features, identified by the DDI-AI transparent heatmaps, significantly increased the odds of correct management and identification of malignant lesions (Table 5). The presence of MD3PC-specific features alone carried the highest odds of revealing an underlying malignancy when identified by the DDI-AI heatmaps (Table 6). 

Similarly, the ROC analysis of the MD3PC feature performance alone demonstrated significant improvements in AUC when comparing DDI (AUC = 61.6%; 95% CI [59.2–64.0%]), DDI-AI (AUC = 67.2%; 95% CI [64.9–69.6%]), and DDI-AI heatmaps (AUC = 75.6%; 95% CI [73.3–77.9%]) in that order (*p* = 0.001244, and *p* < 0.00001, respectively; Figure 3).

The proportion of melanomas correctly managed (chosen for biopsy) by dermatologists was 67.4% (97/144) with clinical images, 86.8% (125/132) with DDI, and increased to 92.4% (133/144) with the DDI-AI device. The number of false negatives for melanoma with clinical images was 47/144, with DDI was 19/144, and decreased to 11/144 with the DDI-AI device. The number of false negatives for severely atypical nevi (including 1 atypical Spitz nevus) managed with clinical images was 41/144, with DDI was 18/144, and decreased to 13/144 with the DDI-AI device.

The interclass correlation (Cohen’s kappa) in management confidence slightly improved from 0.144 when switching from clinical images to DDI and to 0.397 when the DDI-AI device output was added (Table 7). A similar improvement was noted for the confidence in diagnosis (from k = 0.13 to k = 0.388, respectively).

## 4. Discussion

Our results support that the DDI-AI device performs safely and effectively as a stand-alone tool for further evaluation of suspicious and potentially malignant TSCLs that present to a dermatology office. Dermatologists’ use of the DDI-AI device improved both management and diagnostic sensitivity (91.1% and 86.1%, respectively) and specificity (71.0% and 80.7%, respectively). There is a net improvement in detection accuracy with the availability of DDI-AI device output, and its use in patient management significantly improved inherent validity, as reported by area AUC for both diagnosis and management. There was only a slight agreement in dermatologist confidence when using clinical images and DDI, while a fair agreement in confidence was noted when using DDI and the DDI-AI device.

There are several other imaging devices available for clinical use to evaluate concerning skin lesions, all of which are traditionally used in a dermatology office. EIS (Nevisense^®®^, Scibase-AB, Stockholm, Sweden) measures variations in electrical resistance in cells and uses AI to detect their malignant nature [16]. Currently, it is FDA approved for melanoma detection by dermatologists, with a reported sensitivity of 96–98% and specificity of 34.4%. Studies have shown that incorporating EIS alters dermatology trainees’ biopsy decisions by 24.3%, increases sensitivity (80.7% to 95.2%), and improves the biopsy decisions of dermatologists and dermatology providers (physician assistants/associates, nurse practitioners) [29]. ESS (Dermasensor Inc., Miami, FL, USA) is another handheld device that detects MMs, BCCs, SCCs, and AMNUMPs by reflectance optical spectroscopy [30]. It is currently FDA cleared for use by PCPs in patients over 40 years of age to triage concerning skin lesions and refer them to a dermatology office, but it is not labeled for use by dermatologists for stand-alone triage. ESS has been shown to improve melanoma diagnostic sensitivity of PCPs from 67% to 88% (*p* < 0.0001) and management sensitivity from 81% to 94% (*p* = 0.0009) [18,31]. A multispectral optical device (Electro-Optical Sciences, Irvington, NY, USA) was the first FDA-approved device for melanoma detection but was discontinued for clinical use in 2017 due to poor quality of specification. It had reported sensitivity of 98.4% for melanoma, though specificity was 9.9% [19,20]. To our knowledge, the clinical utility of an image-based DDI-AI algorithm with FDA breakthrough designation status has not been tested in a large sample set of 50 TSLs to date.

Therefore, new evidence for use of a safe and effective stand-alone device in clinical settings is high yield, particularly when it may improve objective clinician bedside dermoscopy accuracy using the current dermoscopic standard of care. The diagnostic and management performance reported in this study is superior to other recent FDA-cleared tools and enriches the high degree of trust needed for regulatory recognition and implementation of a new image-based algorithm, SaMD, into new clinical practice settings.

When compared to a recently FDA-cleared AI-based bedside device, though diagnostic and management sensitivity are similar between devices in an identically designed study, diagnostic specificity was 27.7% higher using the DDI-AI device (baseline 73.6% with clinical images to 80.7% with the DDI-AI device) compared to ESS (baseline 40% with clinical images to 53%, respectively). Additionally, the management specificity both increased from baseline and was 40% higher with the DDI-AI device (baseline 64.9% with clinical images to 71% with the DDI-AI device) compared to ESS (baseline 36% with clinical images to 31%, respectively).

This considerable improvement in specificity attests to the importance of dermoscopy use that is widely considered a standard triage tool and emphasized in U.S.-based dermatology, compared to other non-invasive methods currently available (e.g., RCM, OCT, EIS, ESS, PLAs) that typically rely on pre-test probability determination using naked-eye examination (ABCDE). The DDI-AI device provides a fundamental advantage by augmenting the already existing standard of care, resulting in considerable improvement in dermoscopy and triage performance as reported here [13].

Our study contributes novel evidence for a valid stand-alone use of a DDI-AI device that improves safe and effective detection of skin cancers by augmenting current clinical practice where dermatologists are already likely to use a handheld dermatoscope, have a smartphone in their pocket, and/or an office-dedicated tablet available. This context is important when considering the considerable increase in AUC for dermatologist management and diagnosis of TSLCs of concern from a baseline when using the DDI-AI device, which implies that dermatologists using the DDI-AI device would improve their dermoscopy performance and potentially biopsy selection.

### Limitations

There are a few limitations to this study. Although patient ages, clinical images, and DDI were provided to participants (all patient images used in this study were from retrospective chart reviews, and all images were de-identified in accordance with the HIPAA privacy rule), the study design did not allow for a live in-person evaluation of the TSLCs, which is more commonly encountered than a virtual spot (mole) check visit. Importantly, although dermatologists who participated in this study expressed interest in the DDI-AI device, selection bias was minimized by ensuring the participants had a wide range of dermoscopy training and experience. Hence, the strength of this study is that the participants had a diverse and equitable level of clinical experience and approach to evaluation of cases in this study. Future studies that analyze the performance of the DDI-AI device per lesion type may be beneficial.

## 5. Conclusions

In conclusion, the DDI-AI device has the potential to improve bedside decisions made by dermatologists regarding which skin lesions require further evaluation or possible biopsy to rule out skin cancer. This novel technology implementing the standard of care (dermoscopy), augmented by an AI algorithm trained by dermoscopy experts, considerably improves the sensitivity, safety, and effectiveness of skin cancer management. The use of the DDI-AI device by dermatologists is further supported by its ease of use to acquire DDI using existing in-office smartphones or tablets and quick DDI-AI device output, obtained in under 10 s on average when using a strong internet connection. The limited training required to incorporate the device into daily practice and the considerable improvement in diagnostic and management performance make the DDI-AI device a viable tool for daily clinical use in skin cancer detection.

## Figures and Tables

**Table 1:** Characteristics of study cases (*n* = 50).

Characteristic	Total	Benign	Malignant
Number, *n* (%)	50 (100.0%)	25 (100.0%)	25 (100.0%)
Age µ (SD)	60.9 (18.8)	61.0 (18.8)	60.8 (18.6)
Gender			
Male, *n* (%)	34 (68.0%)	16 (64.0%)	18 (72.0%)
Female, *n* (%)	16 (32.0%)	9 (36.0%)	7 (28.0%)
Fitzpatrick skin type, *n* (%)			
I	6 (12.0%)	0 (0.0%)	6 (24.0%)
II	21 (42.0%)	10 (40.0%)	11 (44.0%)
III	14 (28.0%)	11 (44.0%)	5 (20.0%)
IV	7 (14.0%)	4 (16.0%)	3 (12.0%)
Anatomic location, *n* (%)			
Head	12 (24.0%)	4 (16.0%)	8 (32.0%)
Trunk	21 (42.0%)	11 (44.0%)	10 (40.0%)
Upper extremities	10 (20.0%)	7 (28.0%)	3 (12.0%)
Lower extremity	7 (14.0%)	3 (12.0%)	4 (16.0%)
Melanocytic, *n* (%)			
Yes	13 (26.0%)	6 (24.0%)	7 (28.0%)
No	37 (74.0%)	19 (76.0%)	18 (72.0%)
Pigmentation, *n* (%)			
Pigmented	31 (42.6%)	16 (64.0%)	15 (60.0%)
Non-pigmented	19 (18.0%)	9 (36.0%)	10 (40.0%)
Color, *n* (%)			
Dark	17 (34.0%)	7 (28.0%)	10 (40.0%)
Light	21 (42.0%)	13 (52.0%)	8 (32.0%)
Light and dark	12 (24.0%)	5 (20.0%)	7 (28.0%)
Surface, *n* (%)			
Flat	14 (28.0%)	6 (24.0%)	8 (32.0%)
Elevated	36 (72.0%)	19 (76.0%)	17 (68.0%)
Texture, *n* (%)			
Smooth	30 (60.0%)	17(68.0%)	13 (52.0%)
Rough	20 (40.0%)	8 (32.0%)	12 (48.0%)
Biopsied, *n* (%)	34 (68.0%)	9 (36.0%)	25 (100.0%)
Diagnosis, *n* (%)			
Seborrheic keratosis	10 (20.0%)		
Basal cell carcinoma	9 (18.0%)		
Squamous cell carcinoma	8 (16.0%)		
Actinic keratosis	3 (6.0%)		
Melanoma	2 (4.0%)		
Melanoma in situ	2 (4.0%)		
Severely atypical nevus	3 (6.0%)		
Solar lentigo	2 (4.0%)		
Benign melanocytic nevus	1 (2.0%)		
Blue nevus	1 (2.0%)		
Dermatofibroma	1 (2.0%)		
Hemangioma	1 (2.0%)		
LPLK	1 (2.0%)		
Mild atypical nevus	1 (2.0%)		
Sebaceous hyperplasia	1 (2.0%)		
Squamous cell carcinoma in situ	1 (2.0%)		

Assessment whether a study case was melanocytic was determined by two dermatologists concordantly choosing parent pathology classifications into either melanocytic or non-melanocytic. Pigmentation of study case was determined by two dermatologists reviewing clinical images of each lesion and concordantly choosing whether the study case was pigmented. Melanoma, melanoma in situ, basal cell carcinoma, squamous cell carcinoma, atypical melanocytic nevi of uncertain malignant potential (AMNUMPs severely atypical melanocytic nevi) were considered malignant and all others benign; AMNUMPs were considered malignant due to their frequent management by dermatologists with a re-excision (5 mm up to 1 cm margin). 50 cases in this study were collected from 46 patients.

**Table 2:** Participant demographic information (*n* = 36).

Characteristic	Frequency *n* (%)
Gender	
Male	26 (72.2%)
Female	10 (27.8%)
Specialization	
Clinical dermatologist	30 (83.3%)
Dermatopathologist	6 (16.6%)
Years of relevant work experience	
1–5	2 (5.6%)
6–10	12 (33.3%)
11–15	7 (19.4%)
16–20	2 (5.6%)
>20	13 (36.1%)
Type of practice environment	
Academic	16 (44.4%)
Group private practice (dermatology)	14 (38.9%)
Group private practice (multidisciplinary)	2 (5.6%)
Federally qualified health center	2 (5.6%)
Solo private practice	1 (2.8%)
Hospital	1 (2.8%)
Other	1 (2.8%)
Type of population setting	
Urban area	33 (63.6%)
Urban cluster	12 (33.3%)
Rural	1 (2.8%)
Frequency of performing biopsies	
>3 per week	30 (83.3%)
1–3 per week	2 (5.6%)
Few times per year	2 (5.6%)
None	2 (5.6%)
Dermatology referral frequency	
Always	3 (8.3%)
Most of the time	1 (2.8%)
Sometimes	1 (2.8%)
Rarely	5 (13.9%)
Never	26 (72.2%)
Self-rated skin lesion assessment competence	
Intermediate	3 (8.3%)
Advanced	12 (33.3%)
Expert	21 (58.3%)
Perform skin checks	
Yes	34 (94.4%)
No	2 (5.6%)

**Table 3:** Unadjusted sensitivity and specificity of dermatologists with and without the DDI-AI device.

	Modality	B	M	P	N	TP	FN	FP	TN	Total	Sensitivity	95% CI	Specificity	95% CI
Management	Clinical Image Only	900	900	946	854	630	270	316	584	1800	70.0%	[66.9:72.9]	64.9%	[61.7:67.9]
	DDI	900	900	1043	757	759	141	284	616	1800	84.3%	[81.8:86.6]	68.4%	[65.3:71.4]
	DDI-AI Device	900	900	1081	719	820	80	261	639	1800	91.1%	[89.1:92.8]	71.0%	[68.0:73.9]
Diagnosis	Clinical Image Only	900	900	809	991	571	329	238	662	1800	63.4%	[60.2:66.5]	73.6%	[70.6:76.3]
	DDI	900	900	926	874	709	191	217	683	1800	78.8%	[76.0:81.3]	75.9%	[73.0:78.6]
	DDI-AI Device	900	900	949	851	775	125	174	726	1800	86.1%	[83.7:88.2]	80.7%	[78.0:83.1]

DDI = digital dermoscopy image, DDI-AI = DDI-artificial intelligence algorithm, B = benign, M = malignant, P = positive, N = negative, TP = true positive, FN = false negative, FP = false positive, TN = true negative.

**Table 4:** Comparison of management and diagnostic sensitivity, specificity, and ROC with and without DDI-AI.

	Clinical Image	DDI	DDI-AI	* Sig.DDI vs. CI	* Sig.DDI-AI vs. DDI
Management Performance					
Sensitivity [95% CI]	70.0 [66.9:72.9]	84.3 [81.8:86.6]	91.1 [89.1:92.8]		
Specificity [95% CI]	64.9 [61.7:67.9]	68.4 [65.3:71.4]	71.0 [68.0:73.9]		
AUC [95% CI]	69.7 [67.3:72.1]	79.9 [77.8:82.0]	85.0 [83.2:86.8]	<0.00001	0.00033
Diagnostic Performance					
Sensitivity [95% CI]	63.4 [60.2:66.5]	78.8 [76.0:81.3]	86.1 [83.7:88.2]		
Specificity [95% CI]	73.6 [70.6:76.3]	75.9 [73.0:78.6]	80.7 [78.0:83.1]		
AUC [95% CI]	69.1 [66.7:71.6]	79.0 [76.8:71.6]	84.2 [82.3:86.1]	<0.00001	<0.00001

DDI = digital dermoscopy image, DDI-AI = DDI-artificial intelligence algorithm (DDI-AI device), CI = confidence interval, AUC = area under the curve. * *p* values come from a bootstrapped ROC comparisons of AUC values.

**Table 5:** Binary multiple logistic regression analysis of management and diagnostic performance.

	Management Performance Analysis												Diagnostic Performance Analysis											
	Clinical Images			DDI			DDI-AI Device			Heatmaps			Clinical Images			DDI			DDI-AI Device			Heatmaps		
Factor	aOR	95% CI		aOR	95% CI		aOR	95% CI		aOR	95% CI		aOR	95% CI		aOR	95% CI		aOR	95% CI		aOR	95% CI	
Intercept	0.68	[0.40:1.16]		0.31	[0.04:1.85]		0.05	[0.03:0.07]		0.02	[0.00:0.16]		0.72	[0.44:1.20]		0.18	[0.04:0.72]		0.20	[0.02:1.04]		0.37	[0.08:1.35]	
Diagnosis																								
Benign	–			–			–			–			–			–			–			–		
Malignant	4.27	[3.50:5.22]		12.9	[10.2:16.4]		28.8	[21.8:38.4]		9.77	[7.63:12.6]		4.85	[3.96:5.95]		12.0	[9.60:15.0]		26.0	[20.2:33.5]		14.2	[10.1:20.1]	
Confidence in Diagnosis																								
None	–			–			–			–			–			–			–			–		
Slight	0.57	[0.31:1.02]		0.40	[0.07:3.64]		*			3.93	[0.55:79.6]		0.66	[0.38:1.13]		1.55	[0.38:7.19]		0.81	[0.15:7.54]		1.22	[0.32:5.86]	
Moderate	0.61	[0.35:1.07]		0.49	[0.08:4.38]		1.71	[1.10:2.65]		5.90	[0.84:118.7]		0.65	[0.38:1.11]		1.19	[0.30:5.41]		0.78	[0.15:7.16]		0.93	[0.25:4.43]	
High	0.82	[0.47:1.43]		0.88	[0.15:7.78]		3.28	[2.17:4.95]		4.91	[0.70:98.7]		0.74	[0.43:1.27]		1.73	[0.44:7.85]		0.92	[0.17:8.40]		0.38	[0.10:1.83]	
Specific Feature																								
None	–			–			–			–			–			–			–			–		
Asymmetry &/or atypical network										2.30	[1.74:3.04]											1.92	[1.36:2.70]	
Blue-white-grey-violet color(s)										3.62	[2.35:5.66]											2.37	[1.41:4.03]	
Round structures										1.70	[1.24:2.32]											1.36	[0.91:2.01]	

DDI = digital dermoscopy image, DDI-AI = DDI-artificial intelligence algorithm (DDI-AI device), heatmaps = DDI-AI device transparent heatmaps, aOR = adjusted odds ratio. * The reference level was set to “Slight” due to too few cells with confidence = “None”, the model failed to converge. The latter level was removed for the purposes of this model. The models are adjusted for participant confidence in management and diagnosis, with multiple binary logistic models of heatmaps are adjusted for specific features that may have alluded to the suggested management and/or diagnosis.

**Table 6:** Predicting malignancy using MD3PC features.

	DDI		DDI-AI		DDI-AI Heatmaps	
MD3PC Specific Feature	OR	95% CI	OR	95% CI	OR	95% CI
(Intercept)	0.70	[0.61:0.79]	0.50	[0.44:0.58]	0.27	[0.23:0.32]
None	–		–		–	
Asymmetry and/or atypical network	1.92	[1.52:2.44]	3.59	[2.84:4.54]	8.62	[6.63:11.3]
Blue–white–grey–violet color(s)	5.19	[3.61:7.62]	7.44	[4.97:11.42]	13.9	[9.10:21.6]
Round structures	1.48	[1.12:1.95]	1.93	[1.43:2.61]	3.57	[2.59:4.92]

DDI = digital dermoscopy image, DDI-AI = DDI-artificial intelligence algorithm (DDI-AI device), DDI-AI heatmaps = DDI-AI device transparent heatmaps, MD3PC = modified dermoscopy three-point checklist, OR = odds ratio, CI = confidence interval.

**Table 7:** Confidence in management with and without the DDI-AI device output.

		With DDI-AI Device Output				
		None	Slight	Moderate	High	Total
With DDI	None	2	3	1	0	6
	Slight	0	48	80	30	158
	Moderate	0	64	266	213	543
	High	1	30	141	921	1093
	Total	3	145	488	1164	1800

DDI = digital dermoscopy image, DDI-AI = DDI-artificial intelligence algorithm (DDI-AI device). Cohen’s kappa statistic = 0.397, *p* < 0.0001 for inter-rater reliability.

## Data Availability

Original de-identified raw completed survey datasets are available in Supplementary Files provided by a third-party statistician.

## Supplementary Materials

The following supporting information can be downloaded at: https://www.mdpi.com/article/10.3390/cancers16213592/s1, File S1: cancers-16-03592-DDI-AI-analysis.R; File S2: cancers-16-03592-DDI-AI-participant demographics.xlsx; File S3: cancers-16-03592-DDI-AI-participant evaluations.xlsx.

## References

[1] Siegel R.L., Miller K.D., Fuchs H.E., Jemal A. (2021). Cancer statistics, 2021. CA Cancer J. Clin..

[2] Dzwierzynski W.W. (2021). Melanoma Risk Factors and Prevention. Clin. Plast. Surg..

[3] Balch C.M., Soong S.J., Gershenwald J.E., Thompson J.F., Reintgen D.S., Cascinelli N., Urist M., McMasters K.M., Ross M.I., Kirkwood J.M. (2001). Prognostic Factors Analysis of 17,600 Melanoma Patients: Validation of the American Joint Committee on Cancer Melanoma Staging System. J. Clin. Oncol..

[4] Pennie M.L., Soon S.L., Risser J.B., Veledar E., Culler S., Chen S.C. (2007). Melanoma outcomes for Medicare patients: Association of stage and survival with detection by a dermatologist vs. a nondermatologist. Arch. Dermatol..

[5] Rigel D.S., Russak J., Friedman R. (2010). The evolution of melanoma diagnosis: 25 years beyond the ABCDs. CA Cancer J. Clin..

[6] WHAT TO LOOK FOR: ABCDES OF MELANOMA. https://www.aad.org/public/diseases/skin-cancer/find/at-risk/abcdes.

[7] Soyer H.P., Argenziano G., Zalaudek I., Corona R., Sera F., Talamini R., Barbato F., Baroni A., Cicale L., Di Stefani A. (2004). Three-point checklist of dermoscopy. A new screening method for early detection of melanoma. Dermatology.

[8] Zalaudek I., Argenziano G., Soyer H.P., Corona R., Sera F., Blum A., Braun R.P., Cabo H., Ferrara G., Kopf A.W. (2006). Three-point checklist of dermoscopy: An open internet study. Br. J. Dermatol..

[9] Marghoob N.G., Liopyris K., Jaimes N. (2019). Dermoscopy: A Review of the Structures That Facilitate Melanoma Detection. J. Osteopath. Med..

[10] Ferris L.K., Gerami P., Skelsey M.K., Peck G., Hren C., Gorman C., Frumento T., Siegel D.M. (2018). Real-world performance and utility of a noninvasive gene expression assay to evaluate melanoma risk in pigmented lesions. Melanoma Res..

[11] Ludzik J., Becker A.L., Latour E., Lee C., Witkowski A. (2023). Dermoscopic features associated with 3-GEP PLA: LINC00518, PRAME, and TERT expression in suspicious pigmented lesions. Skin. Res. Technol..

[12] Skelsey M., Loftis B., Kaufmann M., Siegel D., Bhatia N., Walker M., Rigby A., Whitaker J., Stone S., Mocia M. (2024). Non-invasive Gene Expression Analysis Rules Out Melanoma with High Negative Predictive Value Regardless of Skin Phototype. SKIN J. Cutan. Med..

[13] Pellacani G., Farnetani F., Ciardo S., Chester J., Kaleci S., Mazzoni L., Bassoli S., Casari A., Pampena R., Mirra M. (2022). Effect of Reflectance Confocal Microscopy for Suspect Lesions on Diagnostic Accuracy in Melanoma: A Randomized Clinical Trial. JAMA Dermatol..

[14] Rajabi-Estarabadi A., Bittar J.M., Zheng C., Nascimento V., Camacho I., Feun L.G., Nasiriavanaki M., Kunz M., Nouri K. (2019). Optical coherence tomography imaging of melanoma skin cancer. Lasers Med. Sci..

[15] Schuh S., Ruini C., Perwein M.K.E., Daxenberger F., Gust C., Sattler E.C., Welzel J. (2022). Line-Field Confocal Optical Coherence Tomography: A New Tool for the Differentiation between Nevi and Melanomas?. Cancers.

[16] Malvehy J., Hauschild A., Curiel-Lewandrowski C., Mohr P., Hofmann-Wellenhof R., Motley R., Berking C., Grossman D., Paoli J., Loquai C. (2014). Clinical performance of the Nevisense system in cutaneous melanoma detection: An international, multicentre, prospective and blinded clinical trial on efficacy and safety. Br. J. Dermatol..

[17] Owji S., Han J., He H., Lopera I., Tassavor M., Brownstone N., Gulati N., Ungar B., Ungar J. (2022). Diagnostic Efficacy of Electrical Impedance Spectroscopy Versus Dermoscopy for Pigmented Skin Lesions: A Pilot Study. SKIN J. Cutan. Med..

[18] Jaklitsch E., Thames T., De Campos Silva T., Coll P., Oliviero M., Ferris L.K. (2023). Clinical Utility of an AI-powered, Handheld Elastic Scattering Spectroscopy Device on the Diagnosis and Management of Skin Cancer by Primary Care Physicians. J. Prim. Care Community Health.

[19] Monheit G., Cognetta A.B., Ferris L., Rabinovitz H., Gross K., Martini M., Grichnik J.M., Mihm M., Prieto V.G., Googe P. (2011). The Performance of MelaFind: A Prospective Multicenter Study. Arch. Dermatol..

[20] Fink C., Jaeger C., Jaeger K., Haenssle H.A. (2017). Diagnostic performance of the MelaFind device in a real-life clinical setting. JDDG J. Dtsch. Dermatol. Ges..

[21] Ludzik J., Becker A.L., Lee C., Witkowski A. (2022). Augmenting pigmented lesion assay results with the three-point dermoscopy checklist to improve pigmented lesion triage. Skin. Res. Technol..

[22] Longo C., Navarrete-Dechent C., Tschandl P., Apalla Z., Argenziano G., Braun R.P., Bataille V., Cabo H., Hoffmann-Wellhenhof R., Forsea A.M. (2023). Delphi Consensus Among International Experts on the Diagnosis, Management, and Surveillance for Lentigo Maligna. Dermatol. Pract. Concept..

[23] Tran T., Cyr P.R., Verdieck A., Lu M.D., Ahrns H.T., Berry E.G., Bowen W., Braun R.P., Cusick-Lewis J.M., Doan H.Q. (2023). Expert Consensus Statement on Proficiency Standards for Dermoscopy Education in Primary Care. J. Am. Board Fam. Med..

[24] Russo T., Piccolo V., Moscarella E., Tschandl P., Kittler H., Paoli J., Lallas A., Braun R.P., Thomas L., Soyer H.P. (2022). Indications for Digital Monitoring of Patients with Multiple Nevi: Recommendations from the International Dermoscopy Society. Dermatol. Pract. Concept..

[25] Gaudy-Marqueste C., Wazaefi Y., Bruneu Y., Triller R., Thomas L., Pellacani G., Malvehy J., Avril M.F., Monestier S., Richard M.A. (2017). Ugly Duckling Sign as a Major Factor of Efficiency in Melanoma Detection. JAMA Dermatol..

[26] Wilson E.B. (1927). Probable Inference, the Law of Succession, and Statistical Inference. J. Am. Stat. Assoc..

[27] Saha K.K., Miller D., Wang S. (2015). A comparison of some approximate confidence intervals for a single proportion for clustered binary outcome data. Int. J. Biostat..

[28] McHugh M.L. (2012). Interrater reliability: The kappa statistic. Biochem. Med..

[29] Litchman G.H., Marson J.W., Svoboda R.M., Rigel D.S. (2020). Integrating Electrical Impedance Spectroscopy into Clinical Decisions for Pigmented Skin Lesions Improves Diagnostic Accuracy: A Multitiered Study. SKIN J. Cutan. Med..

[30] FDA Clearance Granted for First AI-Powered Medical Device to Detect All Three Common Skin Cancers (Melanoma, Basal Cell Carcinoma and Squamous Cell Carcinoma). https://www.dermasensor.com/fda-clearance-granted-for-first-ai-powered-medical-device-to-detect-all-three-common-skin-cancers-melanoma-basal-cell-carcinoma-and-squamous-cell-carcinoma/.

[31] Device Classification Under Section 513(f)(2)(De Novo). https://www.accessdata.fda.gov/scripts/cdrh/cfdocs/cfpmn/denovo.cfm?id=DEN230008.

